# PM_2.5_ Exposure as a Risk Factor for Optic Nerve Health in Type 2 Diabetes Mellitus

**DOI:** 10.3390/toxics12110767

**Published:** 2024-10-22

**Authors:** Tianyi Yuan, Minna Cheng, Yingyan Ma, Haidong Zou, Haidong Kan, Xia Meng, Yi Guo, Ziwei Peng, Yi Xu, Lina Lu, Saiguang Ling, Zhou Dong, Yuheng Wang, Qinping Yang, Wenli Xu, Yan Shi, Cong Liu, Senlin Lin

**Affiliations:** 1 Department of Ophthalmology, Shanghai General Hospital, School of Medicine, Shanghai Jiao Tong University, No. 85/86, Wujin Road, Shanghai 200080, China; yuantianyi@sjtu.edu.cn (T.Y.); mYy_29@163.com (Y.M.); zouhaidong@sjtu.edu.cn (H.Z.); 2 Department of Eye Disease Control and Prevention, Shanghai Eye Disease Prevention and Treatment Center/Shanghai Eye Hospital, No. 1440, Hongqiao Road, Shanghai 200041, China; xxy1982@hotmail.com (Y.X.); lulina1019@163.com (L.L.); 3 Department of Chronic Non-Communicable Diseases and Injury, Shanghai Municipal Centers for Disease Control & Prevention, No. 1380, West Zhongshan Road, Shanghai 200336, China; chengminna@scdc.sh.cn (M.C.); wangyuheng@scdc.sh.cn (Y.W.); yangqinping@scdc.sh.cn (Q.Y.); xuwenli@scdc.sh.cn (W.X.); shiyan@scdc.sh.cn (Y.S.); 4 School of Public Health, Key Lab of Public Health Safety of the Ministry of Education and NHC Key Laboratory of Health Technology Assessment, Fudan University, No. 130, Dong’An Road, Shanghai 200032, China; kanh@fudan.edu.cn (H.K.); mengxia@fudan.edu.cn (X.M.); yguo24@m.fudan.edu.cn (Y.G.); zwpeng24@m.fudan.edu.cn (Z.P.); 5 EVision Technology (Beijing) Co., Ltd., Beijing 100085, China; lingsaiguang@yiweiimage.com (S.L.); dongzhou@yiweiimage.com (Z.D.); 6 National Clinical Research Center for Aging and Medicine, Huashan Hospital, Fudan University, No. 12, Middle Wulumuqi Road, Shanghai 200031, China

**Keywords:** PM_2.5_, optic disc, cup-to-disc ratio, type 2 diabetes mellitus

## Abstract

(1) Objective: This study investigated the relationship between long-term particulate matter (PM_2.5_) exposure and optic disc parameters—vertical cup-to-disc ratio (vCDR), vertical optic disc diameter (vDD), and vertical optic cup diameter (vCD)—in patients with type 2 diabetes mellitus (T2DM). (2) Methods: A cross-sectional analysis was conducted using data from 65,750 T2DM patients in the 2017–2018 Shanghai Cohort Study of Diabetic Eye Disease (SCODE). Optic disc parameters were extracted from fundus images, and PM_2.5_ exposure was estimated using a random forest model incorporating satellite and meteorological data. Multivariate linear regression models were applied, adjusting for confounders including age, gender, body mass index, blood pressure, glucose, time of T2DM duration, smoking, drinking, and physical exercise. (3) Results: A 10 μg/m^3^ increase in PM_2.5_ exposure was associated with significant reductions in vCDR (−0.008), vDD (−42.547 μm), and vCD (−30.517 μm) (all *p*-values < 0.001). These associations persisted after sensitivity analyses and adjustments for other pollutants like O_3_ and NO_2_. (4) Conclusions: Long-term PM_2.5_ exposure is associated with detrimental changes in optic disc parameters in patients with T2DM, suggesting possible optic nerve atrophy. Considering the close relationship between the optic nerve and the central nervous system, these findings may also reflect broader neurodegenerative processes.

## 1. Introduction

The rapid acceleration of global industrialization has increasingly put public health at risk, particularly due to rising levels of air pollution. The long-term health consequences of pollution exposure include poor general health, mental health issues, and even increased risks of fetal, infant, and child mortality [1,2,3]. Fine particulate matter (PM_2.5_) is a significant contributor to this pollution, consisting of solid and liquid particles suspended in the atmosphere with diameters less than 2.5 µm. Major sources of PM_2.5_ include motor vehicle exhaust emissions and the combustion of fuels such as wood, coal, and fuel oil [4]. According to the 2017 Global Burden of Disease Study, PM_2.5_ ranks as the eighth leading cause of mortality worldwide, accounting for 5.25% of all deaths [5]. Numerous epidemiological studies have demonstrated that exposure to PM_2.5_ not only leads to respiratory disorders but also contributes to neurological damage. This damage is primarily mediated through the transmission of pro-inflammatory signals from the liver, lungs, and cardiovascular system to the brain, triggering a cascade of inflammatory responses and oxidative stress. These mechanisms include the release of systemic inflammatory mediators such as interleukin-6 (IL-6), tumour necrosis factor-α (TNF-α), and other pro-inflammatory cytokines, which cross the blood–brain barrier and enter the central nervous system. This process induces microglial activation, increases oxidative stress, and leads to neuronal damage. Consequently, these pathways not only heighten the risk of neurodegenerative diseases, such as Alzheimer’s disease (AD), Parkinson’s disease (PD), and dementia, but also contribute to cognitive impairment and other central nervous system (CNS) disorders [4,6,7,8].

The optic disc, the most forward-extending segment of the optic nerve, represents the anterior end of the central nervous system (CNS). Visual information from the external environment is converted into nerve impulses, which are then transmitted to the retinal ganglion cells through the integration of bipolar cells. The convergence of all axons originating from the retinal ganglion cells (RGCs) at the optic papilla ultimately forms the optic nerve [9]. Routine examination of the optic disc is crucial in preventing nerve-related eye diseases and visual field loss, as changes in the disc’s colour, shape, or depth may indicate underlying pathological changes in the optic nerve. The optic disc is composed of three distinct regions: the neuroretinal rim, the central optic cup, and, in some cases, parapapillary atrophy. Parameters for measuring the optic disc include the diameter of the optic disc, the diameter of the optic cup, and the cup-to-disc ratio (CDR). The CDR is defined as the ratio of the cup diameter (CD) to the disc diameter (DD).

Both type 1 and type 2 diabetes mellitus (T2DM) are strongly associated with reduced cognitive function and anatomical abnormalities in the brain, particularly in domains such as executive function, memory, attention, and nerve conduction velocity. Research has shown that over a period of 3–4 years, patients with T2DM experience a more rapid progression of white matter lesions and brain atrophy compared to non-diabetic controls [10,11]. These lesions are characterized by impaired connectivity, disruption of structural integrity, and increased signal intensity [11]. Since the brain’s white matter is primarily composed of myelinated neuronal fibres, similar to the optic nerve at the optic disc, changes in optic disc parameters—such as the CDR, DD, and CD—in diabetic patients may accurately and promptly reflect changes in cognitive performance. In this study, we aimed to investigate the relationship between PM_2.5_ exposure and optic disc-related parameters by analyzing air pollution levels in the residential areas of 65,750 patients with T2DM with long-term follow-up in the Shanghai community.

## 2. Materials and Methods

### 2.1. Study Population

The population for this study was derived from the Shanghai Cohort Study of Diabetic Eye Disease (SCODE), a cohort study focused on diabetic patients within the Shanghai community, in China (clinicaltrials.gov identifier: NCT03665090). Since 2003, the SCODE study has conducted annual monitoring of both systemic and ocular conditions in diabetic patients across various communities in Shanghai. The inclusion and exclusion criteria, along with the specific methodologies employed, have been thoroughly detailed in previous publications [12,13]. Inclusion criteria were as follows: (1) age above 18 years and (2) presence of clear refractive media. Exclusion criteria included (1) the presence of other ocular diseases, such as glaucoma, macular degeneration, or choroidal diseases, and (2) a history of previous ophthalmic surgeries, such as cataract or retinal surgery. The study adhered to the principles outlined in the Declaration of Helsinki and received approval from the Ethics Committee of Shanghai General Hospital, School of Medicine, Shanghai Jiao Tong University (approval number: 2017KY138). Written informed consent was obtained from each participant. The study also conformed to the Strengthening the Reporting of Observational Studies in Epidemiology (STROBE) guidelines.

In our study analysis, we selected a total of 73,449 participants from patients with T2DM who attended follow-up visits in SCODE 2017-2018. After excluding participants with blurred fundus images (6058) and those with unknown image resolution (1641), we included 65,750 eligible participants in the final analysis. Data were collected through personal interviews, standardized physical examinations, comprehensive ophthalmologic assessments, laboratory tests, and demographic surveys. Demographic characteristics assessed included gender, age, and duration of diabetes. Individual characteristics assessed encompassed body mass index (BMI), smoking and alcohol consumption in the past three months, physical activity duration, fasting blood glucose (FBG), and blood pressure (BP). BMI (kg/m^2^) was calculated as weight (kg) divided by the square of height (m^2^). Physical activity was assessed by multiplying the number of days per week of moderate activity by the number of hours of exercise per session. FBG levels were measured under standardized conditions, before any morning insulin doses. The ophthalmologic examination included visual acuity testing, a slit-lamp examination (66 Vision Tech, Suzhou, China), and fundus photography using a nondilated 45-degree fundus camera (Topcon NW400, Topcon, Tokyo, Japan).

### 2.2. Levels of Air Pollution

**Figure 1:**
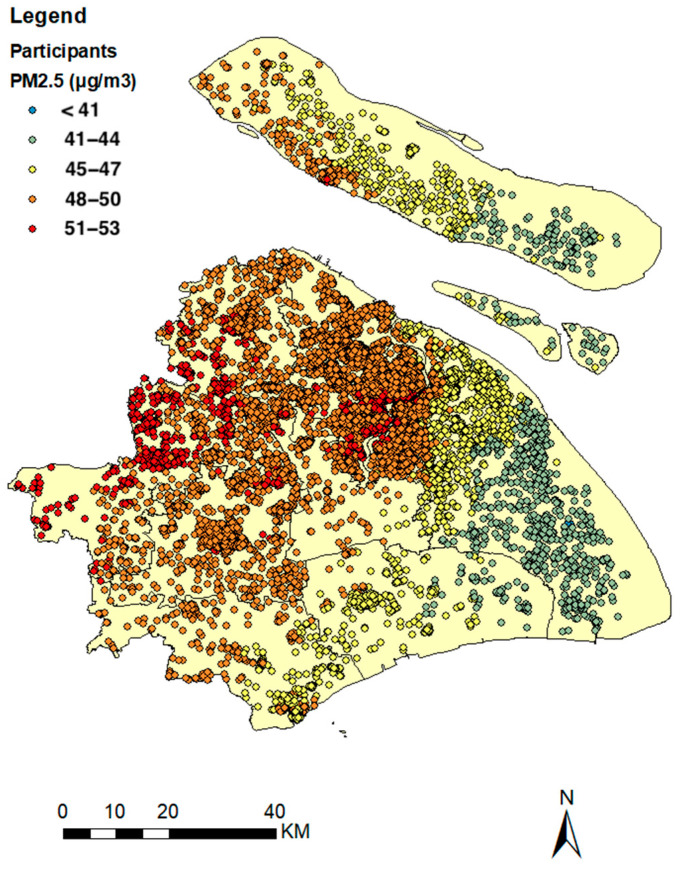
Distribution map of participants’ residential locations and long-term PM_2.5_ concentrations (2013–2017).

We collected PM_2.5_ concentrations at a 1 km^2^ resolution for the years 2013–2017 using a state-of-the-art random forest model, which provided full temporal and spatial coverage of PM_2.5_ [14]. We then matched the corresponding annual average PM_2.5_ levels to the geocodes generated from the home addresses provided by patients during their follow-up visits (see Figure 1). The method combines the satellite-based Multiangle Implementation of Atmospheric Correction (MAIAC) for acquiring high-resolution Aerosol Optical Depth (AOD) models with the NASA-developed MERRA-2 PM_2.5_ model. By incorporating covariates such as temperature, relative humidity, precipitation, total cloudiness, wind direction, and population density, this approach provides accurate, reliable, high-resolution, and comprehensive spatial and temporal coverage of PM_2.5_ exposure levels (R^2^ value of daily average exposure = 0.80; R^2^ value of monthly average exposure = 0.83). We developed a random forest model by combining ground ozone measurements from fixed stations, ozone simulations from the Community Multiscale Air Quality (CMAQ) modelling system, meteorological parameters, population density, road length, and elevation to predict ground maximum daily 8 h average (MDA8) ozone concentrations at a daily level and 1 km × 1 km spatial resolution [14]. Daily NO_2_ concentrations were predicted at a spatial resolution of 1 km in mainland China using random forest models incorporating multiple predictors. Specifically, POMINO-TROPOMI NO_2_ VCD, CMAQ-simulated NO_2_ concentrations, meteorological data, elevation, population, road networks, and NDVI were included [15].

### 2.3. Measurement of Optic Disc Diameters

Optic disc diameter measurements were obtained using an automatic extraction program developed by our team [16]. This method enables direct determination of pixel spacing around the optic disc in fundus images without requiring knowledge of the specific fundus camera or its parameters. Since the average diameter ratio of the selected area to the optic disc remains constant, the true sizes of the optic disc and optic cup can be quickly and accurately measured based on the automatically calculated pixel spacing of the selected area. Given that the optic disc can rotate around the vertical, sagittal, and horizontal axes due to the length of the eye axis, and that discrepancies between two-dimensional fundus photography and three-dimensional reality are typically more pronounced in the horizontal direction than in the vertical [17], we focused on measuring the vertical cup-to-disc ratio (vCDR), vertical optic disc diameters (vDDs), and vertical optic cup diameters (vCDs).

### 2.4. Statistical Analyses

To explore the independent associations between PM_2.5_ and vCDR, vDD, and vCD, we used multivariate weighted linear regression models. The potential covariates in all four models were adjusted stepwise, with the exception of the unadjusted model. Model 1 was adjusted for age, gender, and duration of diabetes; Model 2 was further adjusted for BMI, smoking and drinking status, and physical activity level; Model 3 additionally controlled for BP and FBG; and Model 4 included adjustments for NO_2_ and O_3_ concentrations at the site of residence. Since the effect size associated with a 1 μg/m^3^ change in PM_2.5_ was relatively small and less intuitive to interpret, we rescaled PM_2.5_ to reflect associations per 10 μg/m^3^ increase in the model, to facilitate a clearer interpretation of its relationship with health outcomes. The results of linear regression are represented by the β value and the standard error (SE).

Furthermore, sensitivity analyses were conducted to adjust the modelling of mean PM_2.5_ concentrations across various time horizons and to assess the temporal variability in the relationship between mean PM_2.5_ concentrations over 1, 2, and 3 years and the outcomes. Subsequent subgroup analyses will categorize patients by gender (female or male), age (≤65 or >65 years), BMI (≤24 kg/m^2^ or >24 kg/m^2^), duration of diabetes mellitus, FBG, and blood pressure. This approach aims to evaluate whether the relationship between PM 2.5 exposure and optic nerve parameters (vCDR, vDD, and vCD) is modified by factors such as age, gender, smoking status, and comorbidities. All statistical analyses were performed using the SPSS software, version 26.0 (IBM, New York, NY, USA). A *p*-value of less than 0.05 (two-sided) was considered statistically significant.

## 3. Results

Table 1 displays the baseline characteristics of the 65,750 patients with T2DM from SCODE who underwent examination from 1 January 2017 to 31 December 2017. Of these, 29,470 were males (44.8%) and 36,280 were females (55.2%). The average age of the participants was 64.73 ± 7.53 years, with the duration of DM being 8.01 ± 5.52 years. The patients exhibited good glycemic control, with an FBG level of 6.89 ± 1.31 mmol/L, perhaps due to their annual follow-up at the community hospital’s endocrinology department.

The distribution of optic disc parameters and air pollution exposure in the study participants’ residential locations are presented in Table 2. The interquartile ranges (IQRs) for vCDR, vDD, and vCD were 0.12, 239.18 μm, and 305.03 μm, respectively. Based on the annual average daily air pollutant concentrations at the patients’ primary residences, the IQRs of PM_2.5_, O_3_, and NO_2_ were 2.63, 1.48, and 16.25 μg/m^3^, respectively.

After adjusting for multiple study models, the linear regression results indicated significant decreases in vCDR, vDD, and vCD per 10 μg/m^3^ increase in PM_2.5_ (all β < 0; all *p* < 0.05; coefficients of control variables for all models can be seen in Table 3 and Appendix A). Specifically, vCDR decreased by 0.008 per 10 μg/m^3^ increase in PM_2.5_ (β (SE) = −0.008 (0.002), *p* < 0.001), after controlling for various demographic factors and other air pollutants, such as O_3_ and NO_2_, at the participants’ places of residence. In terms of optic nerve parameters, vDD decreased by 42.547 μm per 10 μg/m^3^ increase in PM_2.5_ (β (SE) = −42.547 (4.406), *p* < 0.001), and vCD decreased by 30.517 μm (β (SE) = −30.517 (5.362), *p* < 0.001).

Given potential variations in air pollution exposure levels from year to year, we conducted sensitivity analyses by adjusting for the mean PM_2.5_ concentrations across different time periods (Table 4). Additionally, after adjusting for the covariates in Model 4—such as age, sex, diabetes duration, BMI, smoking and drinking status, exercise time, BP, FBG, and concentrations of O_3_ and NO_2_ during the corresponding periods—the relationships between one-, two-, and three-year mean PM_2.5_ concentrations and optic disc-related parameters remained consistent with the results of the analyses based on five-year mean concentrations (all β < 0; all *p* < 0.05).

**Figure 2:**
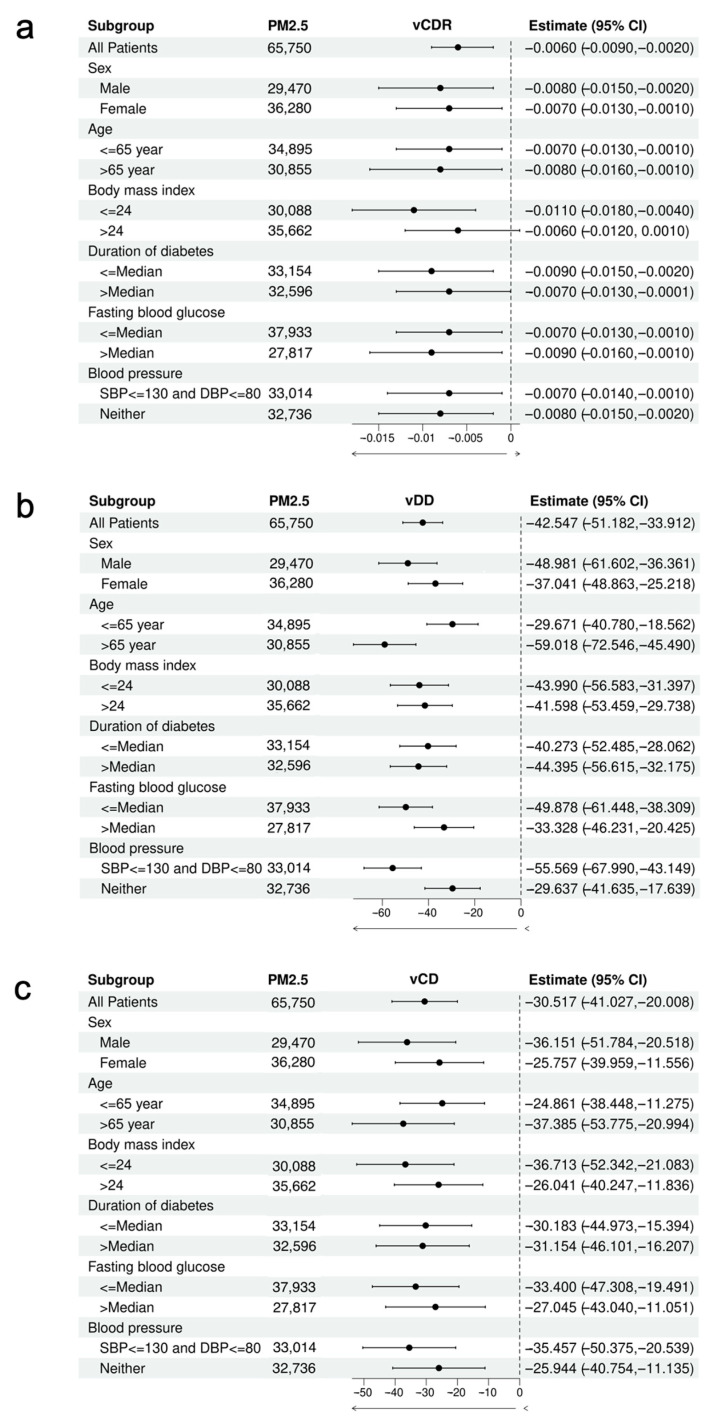
Subgroup analysis of the association between PM_2.5_ and optic disc parameters. The estimates represent the regression coefficient of a 10 μg/m^3^ increase in PM_2.5_ on (**a**) vCDR, (**b**) vDD, and (**c**) vCD. They were calculated for each subgroup using linear regression adjusting for gender, age, gender, body mass index, duration of diabetes, fasting blood glucose, and blood pressure. vCDR, vertical cup-to-disc ratio; vDD, vertical disc diameter; vCD, vertical cup diameter; CI: confidence interval.

Subgroup analyses of the relationship between PM_2.5_ and optic disc parameters, stratified by age, sex, BMI, the duration of DM, FBG, and BP, are presented in Figure 2. The relationships between PM_2.5_ and both vDD and vCD remained consistently stable and negative across all covariate subgroups. For vCDR, the negative correlation with PM_2.5_ remained significant in nearly all covariate subgroups, except for those with a BMI > 24 kg/m^2^ (*p* > 0.05). Moreover, as shown in Figure 2b, patients over 65 years of age experienced a more pronounced decrease in vDD (β (SE) = −59.018 (6.902), *p* < 0.001) compared to those under 65 years (β (SE) = −29.671 (5.668), *p* < 0.001). Additionally, patients with SBP ≤ 130 mmHg and DBP ≤ 80 mmHg also exhibited a greater decrease in vDD (β (SE)= −55.569 (6.337), *p* <0.001).

## 4. Discussion

The relationship between short-term exposure to pollutants and central nervous system damage in patients with DM has been previously established. However, the effects on the optic nerve and optic disc remain unclear. Visual impairment associated with diabetes, such as reduced vision and visual field deficiencies, is receiving increasing attention. This study is the first to investigate the connection between PM_2.5_ exposure and vCDR, vDD, and vCD in patients with T2DM. We discovered a consistent inverse linear association between these parameters by employing several regression models to analyze a sizable cohort. Furthermore, this relationship remained statistically significant when examining subgroups categorized by age, sex, BMI, duration of DM, blood glucose levels, and BP.

As one of the first cities in China to undergo modern industrialization, Shanghai has confronted the negative impacts of this process, including water, air, and soil pollution, while other cities remained in the early stages of development. According to the Shanghai Environmental Bulletin published by the local government, during the year 2018, air quality was classified as “good” on only 78, 58, and 93 days in 2016, 2017, and 2018 [18]. The annual concentration of PM_2.5_ in 2019–2020 reached the secondary National Ambient Air Quality Standards of China (NAAQSCs) for PM_2.5_ (GB 3095–2012, 35 μg/m^3^) for the first time [19]. Numerous studies conducted in Shanghai have demonstrated that elevated PM_2.5_ concentrations significantly increase the incidence of cardiovascular, respiratory, and CNS disorders [19,20,21]. Moreover, patients exposed to higher concentrations of PM_2.5_ may be more susceptible to the adverse effects of diabetes, particularly diabetic complications and mortality [22]. Our research found that for every 10 μg/m^3^ increase in PM_2.5_, there was a decrease of 0.008 in the vCDR, 42.547 μm in the vDD, and 30.517 μm in the vCD in patients with T2DM. This reduction may be attributed to axonal injury at the optic disc, potentially leading to a decrease in the number of nerve fibres, which could indicate optic nerve atrophy [9].

Due to its tiny size, PM_2.5_ can penetrate deep into the lungs, reaching the alveoli where it binds to receptors on the surface of alveolar epithelial cells. This triggers the release of inflammatory factors like IL-6 and TNF-α, which enter the bloodstream, leading to increased systemic inflammation, oxidative stress, and disruption of the blood–brain barrier (BBB) [5,6,23]. Studies have shown that PM_2.5_-induced oxidative stress and inflammation activate intracellular signalling pathways, such as the nuclear factor κB pathway and the mitogen-activated protein kinase pathway, which further amplify the release of inflammatory factors [24]. This heightened systemic inflammation and oxidative stress can harm multiple organs, particularly vulnerable areas of the nervous system, such as the optic nerve.

The optic nerve, which extends from the CNS during embryonic development, shares similarities with the CNS in terms of neural anatomy, function, immune response, and degenerative processes [25]. PM_2.5_ can cross the BBB, infiltrate the CNS, and impact the optic nerve. Kang et al. [26] validated that the BBB-penetrating PM_2.5_ initiates astrogliosis, resulting in slight neuronal loss, microglial infiltration, and differentiation into the pro-inflammatory M1 phenotype by creating PM_2.5_-polluted human brain models. Additional pro-inflammatory mediators and nitric oxide released from M1 microglia exacerbate neuronal damage. This cascade of inflammation leads to synaptic impairment, phosphorylated tau accumulation, and neuronal death [6,26]. In the case of optic nerve damage, microglia usually migrate to the injury site within days, but under PM_2.5_ exposure, these over-activated microglia release excessive inflammatory factors and reactive oxygen species. Rather than aiding in neuronal recovery, they intensify oxidative stress and neuroinflammation in RGCs, further damaging the neurons and axons of the optic nerve [27]. Over an 11-year follow-up period, Gayraud Laure et al. [28] observed that higher levels of PM_2.5_ were significantly associated with a more rapid thinning of the retinal nerve fibre layer and microglial activation. A study conducted in Hong Kong, China, also revealed similar results [29]. Our study primarily focused on the size of the optic disc and optic cup. The activation of microglia by PM_2.5_ may contribute to axonal damage at the optic disc, leading to a decrease in the number of optic nerve fibres and, consequently, a reduction in the sizes of both the optic disc and the optic cup [30].

Subgroup and sensitivity analyses further validated our findings. Notably, in the subgroup analysis, we observed statistically significant variations across different age and blood pressure subgroups. The reduced optic disc size in elderly patients aligns with previous research indicating that most neurodegenerative diseases are associated with aging [31]. During the normal aging process, the optic nerve loses about 0.2–0.3% per year of its axons and 0.5–0.6% per year of the RGC [25]. In addition to neuron loss, aging also causes a decrease in myelin and its production in mice and primates [31]. In patients with hypertension, this condition is often accompanied by increased intracranial pressure and optic disc edema [32]. In our study, patients with SBP > 130 mmHg or DBP > 80 mmHg experienced a relatively smaller decrease in optic disc diameter. This may be due to hypertension-induced vasculopathy, which can cause thickening of the vessel walls, peripapillary vascular congestion, and optic disc leakage, leading to optic disc edema that obscures the true optic disc diameter. Our sensitivity analysis showed a more pronounced difference between the 1-year and 3-year averages, likely due to short-term pollution fluctuations, which are less reliable for assessing chronic exposure. In contrast, the 2-year, 3-year, and 5-year averages were more consistent and better reflected long-term pollution levels, which aligns with our study’s focus on the chronic effects of air pollution.

To the best of our knowledge, this is the first clinical study to investigate the relationship between air pollution and the optic disc diameter and cup-to-disc ratio in patients with T2DM. Utilizing a large sample size, we combined a random forest model with artificial intelligence techniques, and gradually adjusted the analytical model to better control potential confounding factors. Sensitivity analyses were also conducted to further confirm the stability of our results. However, our study has some limitations. For instance, we did not exclude the effects of confounding factors such as the degree of myopia and ocular axis length. Additionally, previous research has shown that the rotation angle of the optic disc can influence its diameter. For example, in cases of high myopia, the optic disc’s maximum diameter is often sagittally rotated, with the upper optic disc tilting poleward toward the macula [33], potentially leading to measurement errors in the optic cup and optic disc diameter. Furthermore, we did not account for socio-economic factors such as income and education level, which may influence both health outcomes and exposure to environmental pollutants. Lastly, the cross-sectional nature of our study limits our ability to infer causality between PM_2.5_ exposure and optic nerve or CNS degeneration, highlighting the need for longitudinal analyses to further investigate these associations.

## 5. Conclusions

Given the anatomical and pathophysiological similarities between the optic nerve and CNS, optic nerve imaging can be employed as a noninvasive method for monitoring neurodegenerative brain diseases. Our study found that long-term exposure to PM_2.5_ was associated with reduced optic cup size, optic disc diameter, and CDR in patients with diabetes, as observed through noninvasive fundus photography. These findings suggest that air pollution may induce optic nerve atrophy and degenerative changes in the CNS. Therefore, it is crucial for patients with diabetes living in areas with severe air pollution to undergo regular funduscopic examinations to detect and prevent visual impairment and CNS disorders in a timely manner.

However, given the cross-sectional design of our study, it is not possible to establish a causal relationship between PM_2.5_ exposure and optic nerve or CNS degeneration. Future longitudinal research is needed to confirm these associations and further explore the underlying mechanisms. Such studies will be essential to clarify the temporal relationship between chronic air pollution and neurodegenerative processes. Moreover, future research should explore the specific pathways through which air pollution contributes to optic nerve atrophy, focusing on mechanisms such as systemic inflammation, oxidative stress, and neuronal damage. The integration of advanced imaging techniques and biomarker analyses would provide valuable insights into the progression of these effects and help inform interventions for high-risk populations, such as individuals with diabetes.

## Figures and Tables

**Table 1:** Descriptive statistics of the study population (n = 65,750).

	Mean ± SD	Min	Max
Male (%)	29,470 (44.82)		
Age (years)	64.73 ± 7.53	18	97
Duration of diabetes (years)	8.01 ± 5.52	0	30
BMI (kg/m^2^)	24.52 ± 3.01	11.72	57.42
SBP (mmHg)	129.55 ± 9.09	84	220
DBP (mmHg)	78.42 ± 6.12	30	122
FBG (mmol/L)	6.89 ± 1.31	2.2	25.9
Cigarette smoking (%)	5050 (7.68)		
Alcohol drinking (%)	3056 (4.65)		
Physical exercise (min/week)	136.58 ± 128.13	0	500

SD, standard deviation; DM, diabetes mellitus; BMI, body mass index; SBP, systolic blood pressure; DBP, diastolic blood pressure; FBG, fasting blood glucose.

**Table 2:** Baseline optic disc diameter and exposure levels to PM_2.5_, O_3_, and NO_2_ from 2013 to 2017.

Variables	Mean ± SD	Percentile				
		25th	Median	75th	Max	IQR
vCDR	0.48 ± 0.1	0.42	0.48	0.54	0.97	0.12
vDD (μm)	1893.34 ± 210.64	1768.69	1885.42	2007.87	3448.8	239.18
vCD (μm)	916.08 ± 243.23	757.87	900.78	1062.9	2549.07	305.03
Air pollution						
PM_2.5_ (μg/m^3^)	47.88 ± 2.25	46.75	48.64	49.38	53.9	2.63
O_3_ (μg/m^3^)	41.72 ± 1.28	41.06	42.22	42.54	44.8	1.48
NO_2_ (μg/m^3^)	22.91 ± 9.41	14.54	23.82	30.79	45.1	16.25

SD, standard deviation; IQR, interquartile range; vCDR, vertical cup-to-disc ratio; vDD, vertical disc diameter; vCD, vertical cup diameter.

**Table 3:** Increments in optic disc diameter associated with 10 μg/m^3^ increase in PM_2.5_.

	Model 1 β (SE)	Model 2 β (SE)	Model 3 β (SE)	Model 4 β (SE)
vCDR	−0.005 (0.002) *	−0.005 (0.002) *	−0.005 (0.002) *	−0.008 (0.002) ***
vDD (μm)	−47.794 (3.837) ***	−48.755 (3.868) ***	−48.262 (3.875) ***	−42.547 (4.406) ***
vCD (μm)	−26.815 (4.669) ***	−27.105 (4.707) ***	−26.988 (4.716) ***	−30.517 (5.362) ***

Model 1 adjusted for age, gender, and DM duration. Model 2: Model 1 + BMI, alcohol consumption, smoking status, and time of physical exercise. Model 3: Model 2 + blood pressure and glucose. Model 4: Model 3 + O_3_ and NO_2_. SE, standard error; vCDR, vertical cup-to-disc ratio; vDD, vertical disc diameter; vCD, vertical cup diameter. * *p* < 0.05, *** *p* < 0.001.

**Table 4:** Sensitivity analyses of alternative PM_2.5_ exposure levels using 1-year, 2-year, and 3-year averages.

Variables	1-Year Average PM_2.5_ β (SE)	2-Year Average PM_2.5_ β (SE)	3-Year Average PM_2.5_ β (SE)
vCDR	−0.005 (0.002)	−0.012 (0.003)	−0.011 (0.003)
vDD (μm)	−28.558 (4.197)	−42.095 (6.324)	−49.725 (5.509)
vCD (μm)	−19.658 (5.111)	−38.589 (7.694)	−39.043 (6.704)

Increment in optic disc diameter associated with 10 μg/m^3^ increase in PM_2.5_ using different exposure metrics. SE, standard error; vCDR, vertical cup-to-disc ratio; vDD, vertical disc diameter; vCD, vertical cup diameter.

## Data Availability

The data that support the findings of this study are available from the corresponding author upon reasonable request.

## Appendix A. Linear Regression Results of the Models

**Table A1:** Increments in vertical cup-to-disc ratio associated with control variables.

	Model 1 β (SE)	Model 2 β (SE)	Model 3 β (SE)	Model 4 β (SE)
PM_2.5_ (per 10 µg/m^3^ increase)	−0.005 (0.002) *	−0.005 (0.002) *	−0.005 (0.002) *	−0.008 (0.002) ***
Age (per year increase)	1.98 × 10^−4^ (6.3 × 10^−5^) **	1.82 × 10^−4^ (6.4 × 10^−5^) **	1.84 × 10^−4^ (6.4 × 10^−5^) **	1.71 × 10^−4^ (6.4 × 10^−5^) **
Gender (male vs. female)	0.016 (0.001) ***	0.016 (0.001) ***	0.016 (0.001) ***	0.016 (0.001) ***
Duration of diabetes (per year increase)	0.0002 (0.0001) *	0.0002 (0.0001)	0.0002 (0.0001)	0.0001 (0.0001)
BMI (per unit increase)		−0.001 (0.0002) ***	−0.001 (0.0002) ***	−0.001 (0.0002) ***
Alcohol drinking (yes vs. no)		−0.002 (0.003)	−0.002 (0.003)	−0.002 (0.003)
Cigarette smoking (yes vs. no)		0.002 (0.002)	0.002 (0.002)	0.002 (0.002)
Physical exercise (per unit increase)		−1.75 × 10^−^⁶ (4.00 × 10^−^⁶)	−1.68 × 10^−^⁶ (4.00 × 10^−^⁶)	−1.59 × 10^−^⁶ (4.00 × 10^−^⁶)
Fasting blood glucose (per unit increase)			0.002 (0.004)	0.002 (0.004)
Blood pressure (per mmHg increase)			−5.67 × 10^−^⁶ (7.6 × 10^−5^)	−3.48 × 10^−^⁶ (7.6 × 10^−5^)
O_3_ (per 10 µg/m^3^ increase)				0.012 (0.005) *
NO_2_ (per 10 µg/m^3^ increase)				4.84 × 10^−4^ (0.001)

SE, standard error. * *p* < 0.05, ** *p* < 0.01, *** *p* < 0.001.

**Table A2:** Increments in vertical optic disc diameters associated with control variables.

	Model 1 β (SE)	Model 2 β (SE)	Model 3 β (SE)	Model 4 β (SE)
PM_2.5_ (per 10 µg/m^3^ increase)	−47.794 (3.837) ***	−48.755 (3.868) ***	−48.262 (3.875) ***	−42.547 (4.406) ***
Age (per year increase)	−1.114 (0.117) ***	−1.031 (0.118) ***	−1.046 (0.118) ***	−1.019 (0.118) ***
Gender (male vs. female)	8.048 (1.738) ***	4.966 (1.838) **	5.037 (1.839) **	5.213 (1.84) **
Duration of diabetes (per year increase)	−0.139 (0.159)	−0.112 (0.16)	−0.07 (0.161)	−0.048 (0.161)
BMI (per unit increase)		0.578 (0.288) *	0.619 (0.29) *	0.591 (0.291) *
Alcohol drinking (yes vs. no)		−3.172 (4.706)	−3.277 (4.707)	−2.708 (4.712)
Cigarette smoking (yes vs. no)		−17.318 (3.831) ***	−17.614 (3.835) ***	−16.807 (3.846) ***
Physical exercise (per unit increase)		0.005 (0.007)	0.005 (0.007)	0.005 (0.007)
Fasting blood glucose (per unit increase)			−1.653 (0.676) *	−1.691 (0.676) *
Blood pressure (per mmHg increase)			0.045 (0.14)	0.04 (0.14)
O_3_ (per 10 µg/m^3^ increase)				−24.308 (8.92) **
NO_2_ (per 10 µg/m^3^ increase)				−22.143 (1.587) ***

SE, standard error. * *p* < 0.05, ** *p* < 0.01, *** *p* < 0.001.

**Table A3:** Increments in vertical optic cup diameters associated with control variables.

	Model 1 β (SE)	Model 2 β (SE)	Model 3 β (SE)	Model 4 β (SE)
PM_2.5_ (per 10 µg/m^3^ increase)	−26.815 (4.669) ***	−27.105 (4.707) ***	−26.988 (4.716) ***	−30.517 (5.362) ***
Age (per year increase)	0.033 (0.142)	0.037 (0.143)	0.033 (0.143)	0.016 (0.144)
Gender (male vs. female)	32.056 (2.115) ***	30.871 (2.237) ***	30.9 (2.239) ***	30.791 (2.24) ***
Duration of diabetes (per year increase)	0.309 (0.194)	0.271 (0.195)	0.282 (0.196)	0.268 (0.196)
BMI (per unit increase)		−1.282 (0.351) ***	−1.266 (0.354) ***	−1.248 (0.354) ***
Alcohol drinking (yes vs. no)		−4.246 (5.727)	−4.292 (5.729)	−4.644 (5.735)
Cigarette smoking (yes vs. no)		−3.482 (4.663)	−3.582 (4.667)	−4.08 (4.681)
Physical exercise (per unit increase)		−0.001 (0.008)	−0.001 (0.008)	−0.001 (0.008)
Fasting blood glucose (per unit increase)			−0.451 (0.822)	−0.427 (0.822)
Blood pressure (per mmHg increase)			−0.009 (0.17)	−0.007 (0.17)
O_3_ (per 10 µg/m^3^ increase)				15.013 (10.856)
NO_2_ (per 10 µg/m^3^ increase)				−7.327 (1.935) ***

SE, standard error. *** *p* < 0.001.
